# Synergistic Effects of *Paenibacillus polymyxa* NBmelon-1 Inoculation and Grafting Restructure of Rhizosphere Microbiome and Enhanced Disease Resistance in Melon Self-Rootstocks

**DOI:** 10.3390/microorganisms13061172

**Published:** 2025-05-22

**Authors:** Wenjie Dong, Quanyu Zang, Yuhong Wang, Erlei Ma, Weihong Ding, Leiyan Yan, Fangmin Hao

**Affiliations:** 1 Ningbo Key Laboratory of Characteristic Horticultural Crops in Quality Adjustment and Resistance Breeding, Ningbo Academy of Agricultural Sciences, Ningbo 315040, China; dongwenjie032020@163.com (W.D.); zqy0711@163.com (Q.Z.); yhwangsc@163.com (Y.W.); mel1984@126.com (E.M.); dwh21@163.com (W.D.); yanleiyan@zju.edu.cn (L.Y.); 2 College of Biology and Environment, Zhejiang Wanli University, Ningbo 315199, China

**Keywords:** grafting, PGPR, synergistic effects, rhizosphere microbiome, soil-borne disease control

## Abstract

Rhizosphere microorganisms play pivotal roles in mitigating the challenges associated with continuous cropping in melon cultivation. While grafting and plant growth-promoting rhizobacteria (PGPR) independently influence rhizosphere microbial communities, their combined effects remain largely unexplored. This study investigates the synergistic regulation of *Paenibacillus polymyxa* NBmelon-1 inoculation and grafting on rhizosphere microbiome assembly, plant performance, and disease resistance in melon self-rootstocks. Field experiments demonstrated that NBmelon-1 inoculation significantly enhanced rootstock stem diameter (95.3% increase in spring) and root development, achieving a graft survival rate exceeding 95%. The combined treatment (NB+GJ) increased scion fruit yield by 29.8% in autumn and 36.5% in spring, as well as the single-fruit weight by 22.5% in autumn and 37.3% in spring, while maintaining fruit morphology. Integrated 16S rRNA and ITS sequencing revealed that the NB+GJ treatment selectively enriched antagonistic bacterial phyla (e.g., Firmicutes and Actinobacteriota) and suppressed pathogenic fungi (e.g., *Fusarium* and Melanconiella). Seasonal shifts in microbial diversity and functional gene profiles underscored the dynamic interplay between treatments and environmental factors. These findings establish a novel strategy for optimizing melon self-rootstock grafting systems and sustainably managing soil-borne diseases.

## 1. Introduction

*Cucumis melo* L., an annual trailing herb of the Cucurbitaceae family, represents a globally significant horticultural crop valued for its short growth cycle and high economic returns. China, with its extensive cultivation history and vast production regions, has emerged as the world’s largest producer of melons and watermelons in terms of both cultivated area and yield [1]. However, intensive monoculture practices have exacerbated continuous cropping obstacles, particularly the proliferation of soil-borne diseases such as Fusarium wilt caused by *Fusarium oxysporum* f. sp. *melonis*. These pathogens persist in soil as dormant propagules, rapidly colonizing root systems under favorable environmental conditions and causing vascular wilting, yield losses exceeding 50%, and long-term soil degradation [2]. While chemical pesticide provides rapid pathogen suppression, its indiscriminate action disrupts beneficial microbiota, accelerates pesticide resistance, and raises ecological concerns, underscoring the urgent need for sustainable alternatives.

Grafting onto disease-resistant rootstocks has emerged as a cornerstone strategy to mitigate soil-borne pathogens while circumventing chemical inputs [3]. This technique operates through dual mechanisms: (1) exploiting the innate resistance of rootstocks to restrict pathogen invasion, and (2) modulating root exudate profiles to enrich defense-related metabolites such as pathogenesis-related proteins [4,5]. Notably, this occurs with graft-induced systemic acquired resistance in cucurbits, which correlate with the activation of stress-responsive proteins [5]. However, conventional heterografting using pumpkin (*Cucurbita moschata*) or bottle gourd (*Lagenaria siceraria*) rootstocks frequently compromises fruit quality attributes—particularly sugar content and aroma profiles—due to physiological incompatibility between scion and rootstock. In contrast, melon self-grafting (homografting) preserves fruit sensory characteristics and achieves superior graft compatibility, with survival rates exceeding 90% [6,7]. Nevertheless, melon self-rootstocks exhibit intrinsic limitations, including shallow root architecture, reduced nutrient uptake efficiency, and diminished resilience against abiotic stresses, which collectively constrain their commercial adoption.

Plant growth-promoting rhizobacteria (PGPR), particularly members of the genus *Paenibacillus*, offer a promising solution to augment rootstock performance through multifaceted mechanisms. *Paenibacillus polymyxa* suppresses phytopathogens via niche competition, antibiosis (e.g., fusaricidin production), and induction of systemic resistance, while concurrently enhancing plant growth through nitrogen fixation, phosphate solubilization, and phytohormone synthesis. For instance, *P. polymyxa* S3 inoculation increased root length in cowpea (*Vigna unguiculata*) by 21% through plant growth–promoting factors [8]. Our prior work isolated *P. polymyxa* NBmelon-1 from melon rhizospheres, demonstrating its efficacy in suppressing *Fusarium* wilt by 52.27% while increasing plant biomass under greenhouse conditions [9]. However, the potential synergy between PGPR inoculation and grafting remains unexplored in melon cultivation systems.

The rhizosphere, frequently characterized as the micro-domain soil region between plant root systems and the soil environment, constitutes a dynamic interface governing nutrient cycling, stress tolerance, and pathogen antagonism [10,11,12]. Numerous microorganisms inhabit the rhizosphere, and their assembly is shaped by agricultural practices such as grafting, which alters root exudate composition to recruit beneficial taxa like *Bacillus* and *Pseudomonas* while excluding pathogens [13]. For example, grafted watermelon rootstocks enrich *Streptomyces* populations and increase defense-related metabolites such as organosulfur compounds, which effectively inhibit *Fusarium oxysporum* [14]. Similarly, PGPR inoculation can displace indigenous microbial communities, creating pathogen-suppressive soil environments [15]. Despite these advances, critical knowledge gaps persist regarding the following: (1) how grafting and PGPR synergistically reshape melon rhizosphere microbiomes under field conditions, and (2) whether such interventions durably suppress soil-borne pathogens across seasonal cycles.

Here, we hypothesize that pre-grafting inoculation of melon self-rootstocks with *P. polymyxa* NBmelon-1 will synergize with grafting to reconfigure rhizosphere microbial networks, enhance root system vigor, and amplify systemic disease resistance. Through replicated field trials integrating physiological measurements, high-throughput sequencing, and functional genomics, this study aims to complete the following: 1. quantify the agronomic impacts of NBmelon-1 inoculation on melon rootstock development and graft success. 2. characterize treatment-driven shifts in rhizosphere microbial diversity, taxonomic composition, and functional potential. 3. elucidate seasonal dynamics of pathogen suppression and microbiome stability. Our findings establish a novel framework for optimizing melon self-rootstock systems through microbiome engineering, offering a sustainable pathway to mitigate continuous cropping obstacles while preserving fruit quality.

## 2. Materials and Methods

### 2.1. Microbial Strains, Plant Materials, and Experimental Design

The *Paenibacillus polymyxa* strain NBmelon-1 [9], previously isolated from melon rhizosphere soil and characterized for its antagonistic activity against *Fusarium oxysporum*, was provided by the Vegetable Research Institute of Ningbo Academy of Agricultural Sciences (Ningbo, Zhejiang Province, China). Melon rootstock (*Cucumis melo* var. *makuwa* cv. ‘Zhentian No.1’) and scion (*C. melo* var. *inodorus* cv. ‘Baimei No.1’) seeds were obtained from the same institution. Field trials were conducted in a greenhouse at the Ningbo High-Tech Agricultural Technology Experimental Zone (29°41′22″ N, 121°36′32″ E), characterized by a subtropical monsoon climate (annual mean temperature: 16.2 °C; precipitation: 1374 mm).

Rootstock seeds were germinated in 50-cell trays containing a sterile substrate mixture (peat/vermiculite/perlite = 2:1:1, *v*/*v*/*v*). Three treatments were implemented: 1. NB+GJ: Rootstocks were inoculated with 2 mL of NBmelon-1 suspension (108 CFU/mL) at 1, 3, 5, and 7 days post-sowing, followed by grafting. 2. GJ: Rootstocks received equivalent volumes of sterile water before grafting. 3. CK: Non-grafted seedlings served as controls.

Grafting was performed using the hole insertion method when rootstocks reached the first true leaf stage (12–14 days post-sowing) and scions had partially expanded cotyledons. Grafted seedlings were acclimatized in a climate-controlled chamber (25 ± 1 °C, 85% relative humidity, 12 h photoperiod) for 7 days before transplanting to field plots. Each treatment comprised three randomized plots (25 m^2^ per plot, 30 plants/plot) with 40 cm spacing between plants. Trials were replicated during autumn (September 2023) and spring (March 2024) growing seasons.

### 2.2. Phenotypic Trait Measurements

Rootstock morphological parameters (stem diameter, leaf dimensions, root length, and root number) were evaluated 9 days after sowing. Scion growth traits (stem diameter, cotyledon size, largest true leaf length/width) were measured 15 days post-grafting. Graft survival rates were assessed 7 days after transplanting. At harvest, 10 fruits per treatment were randomly selected for quality analysis. Fruit weight, shape index (longitudinal diameter/transverse diameter), and flesh thickness (averaged from top, middle, and bottom regions) were recorded. Soluble solids content (SSC) was quantified using a handheld refractometer (PAL-1, Atago, Tokyo, Japan), while soluble sugars and vitamin C were analyzed via anthrone [16] and 2,6-dichloroindophenol titration methods [17], respectively.

### 2.3. Rhizosphere Soil Sampling and DNA Sequencing

Rhizosphere soil samples (0–20 cm depth) were collected from three randomly selected plants per treatment during fruit maturation. After gently removing loosely adhered soil, samples were sieved (2 mm mesh), homogenized, and stored at −80 °C. Total genomic DNA was extracted using the MagicPure^®^ Soil DNA Kit (TransGen Biotech, Beijing, China) following manufacturer protocols. DNA quality (A260/A280: 1.8–2.0) was verified via NanoDrop™ K5600 (Thermo Fisher, Waltham, MA, USA) and agarose gel electrophoresis.

The V3–V4 hypervariable regions of bacterial 16S rRNA genes were amplified with universal primers 338F (5′-ACTCCTACGGGAGGCAGCA-3′) and 806R (5′-GGACTACHVGGGTWTCTAAT-3′) [18]. Fungal ITS1 regions were targeted using primers ITS1F (5′-CTTGGTCATTTAGAGGAAGTAA-3′) and ITS2R (5′-GACGCTTCTCCAGACTACAAT-3′) [19]. Sequencing libraries were constructed following Illumina’s standard protocols and subjected to paired-end sequencing (2 × 250 bp) on the NovaSeq 6000 platform through commercial service provided by Jisi Huiyuan Biotechnology Co., Ltd. (Nanjing, China). Raw sequences were processed using the DADA2 pipeline to eliminate low-quality reads and chimeric sequences. Paired-end reads were assembled via pandaseq 2.11 software [20], followed by rigorous quality control using PRINSEQ [21]. Amplicon Sequence Variants (ASVs) were subsequently clustered and taxonomically annotated through comparative alignment against the SILVA 132 and UNITE reference databases.

### 2.4. Statistical Analysis

All growth parameters of rootstocks, scions, and fruit quality indices were systematically processed using Microsoft Excel 2023. Significant differences among treatments were determined through one-way ANOVA followed by Duncan’s multiple range test (*p* < 0.05) in IBM SPSS Statistics 19.0. Microbial α-diversity (Shannon and Chao1 indices) and β-diversity (non-metric multidimensional scaling based on Bray–Curtis distance) were calculated using QIIME2. Functional prediction of 16S rRNA sequencing data was conducted via PICRUSt2, with comparative annotation against the Kyoto Encyclopedia of Genes and Genomes (KEGG) and Clusters of Orthologous Groups (COG) databases, yielding abundance profiles of KEGG Orthology (KO) terms and COG functional categories. Circos plots and heatmaps were generated using the “ggplot2” and “pheatmap” packages in R 4.5.0.

## 3. Results

### 3.1. Effects of Different Treatments on Growth of Melon Rootstock and Scion

**Figure 1:**
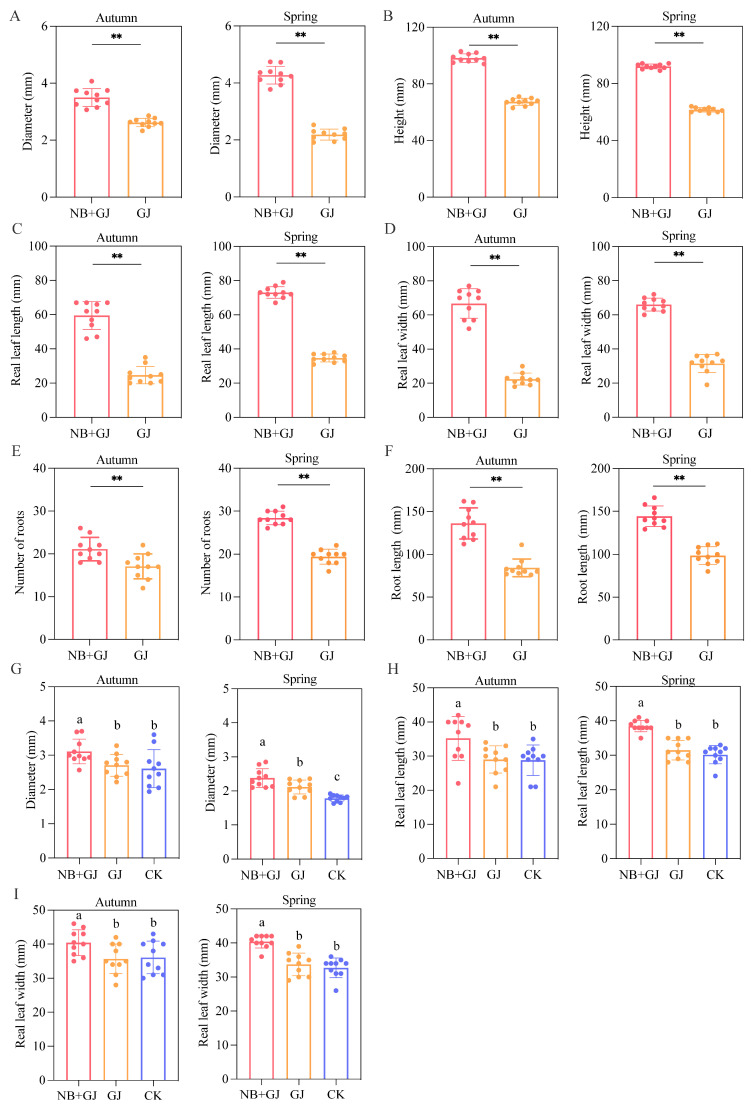
Growth responses of melon rootstock and scion to *P. polymyxa* NBmelon-1 inoculation. Panels (**A**–**F**) quantify rootstock parameters: (**A**) Stem diameter. (**B**) Plant height. (**C**) Length of the largest true leaf. (**D**) Width of the largest true leaf. (**E**) Number of roots. (**F**) Root length. Asterisks denote statistical significance (** *p* < 0.01). Panels (**G**–**I**) illustrate scion responses to inoculation; (**G**) Stem diameter. (**H**) Length of the largest true leaf. (**I**) Width of the largest true leaf. Distinct lowercase letters indicate significant intergroup differences (*p* < 0.05).

Under identical greenhouse conditions, melon rootstocks were treated with either *P*. *polymyxa* NBmelon-1 (NB+GJ) or water (GJ). Compared to the GJ group, inoculation with NBmelon-1 significantly enhanced multiple growth traits of the rootstocks. In the autumn trial, the stem diameter, plant height, and length-to-width ratio of the largest true leaf in the inoculated rootstocks increased by 33.6%, 45.9%, 141%, and 198%, respectively. Corresponding increases in the spring trial reached 95.3%, 49.4%, 110%, and 109% (Figure 1A–D), with both seasonal cohorts exhibiting statistically significant differences compared to the controls. In comparison to the GJ group, the NB+GJ group significantly enhanced the number and length of rootstock roots by 23.4% and 61.4%, respectively, in autumn. The improvements were even more pronounced in spring, with increases of 46.4% and 46.3% (Figure 1E,F). These results indicate that NBmelon-1 inoculation promotes rootstock growth, particularly enhancing root system development. This intervention effectively mitigates the inherent weaknesses of melon rootstock root systems, thereby optimizing subsequent grafting operations.

Three experimental groups were established using melon rootstocks subjected to distinct treatments: (1) inoculation with *P*. *polymyxa* NBmelon-1 (NB+GJ), (2) water-treated rootstocks (GJ), and (3) non-grafted seedling controls (CK). In the grafted groups, the NB+GJ-treated scions exhibited significantly superior growth metrics compared to their GJ-treated counterparts. Relative to the GJ groups, the autumn NB+GJ grafts demonstrated increases of 14.9%, 21.4%, and 13.3% in scion stem diameter, cotyledon length/width, and the dimensions of the largest true leaf, respectively. Corresponding enhancements in the spring NB+GJ grafts reached 12.4%, 22.2%, and 19.9% (*p* < 0.05; Figure 1G–I). While all grafted groups outperformed the CK in scion traits, not all differences achieved statistical significance (*p* > 0.05).

Notably, the NB+GJ group achieved autumn and spring survival rates of 96.0% and 98.5%, respectively, representing significant improvements of 11.0% and 16.0% over the GJ group (Table A1). These results demonstrate that the synergistic effect of NBmelon-1 inoculation and grafting indirectly promotes scion growth and significantly increases the survival rate of grafted seedlings.

### 3.2. Effects of Different Treatments on Fruit Quality and Yield

**Figure 2:**
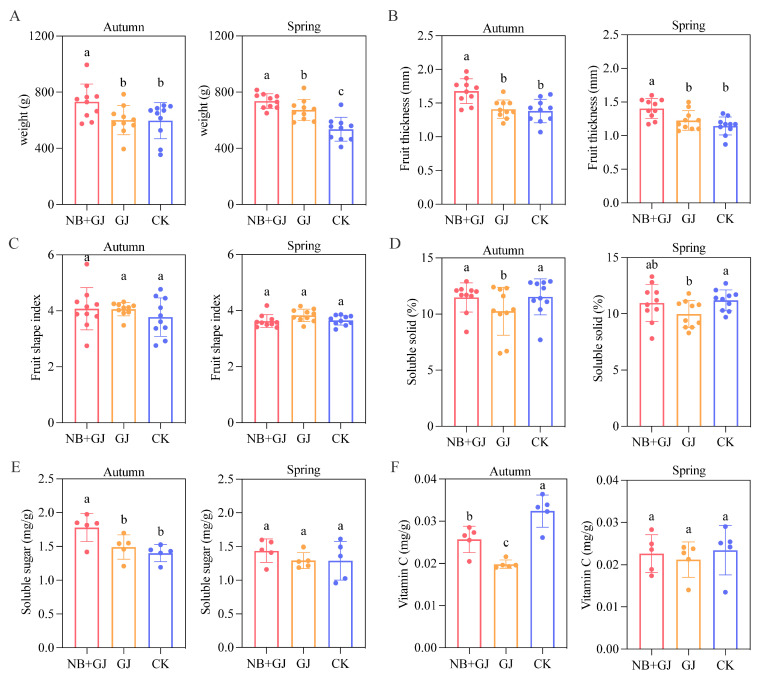
Effects of treatments on melon fruit development. (**A**) Single-fruit weight. (**B**) Fruit thickness. (**C**) Fruit shape index. (**D**) Soluble solids content. (**E**) Soluble sugar content. (**F**) Vitamin C content. Different lowercase letters indicate significant differences (*p* < 0.05).

Treatment modalities had distinct impacts on fruit yield and quality parameters in grafted scions. Compared to the control group (CK), all grafted groups (NB+GJ and GJ) exhibited an upward trend in total fruit yield (Table A2). The NB+GJ group showed a significant increase in single-fruit weight and flesh thickness compared to both the GJ and CK groups (Figure 2A,B). However, the fruit shape index did not reveal any significant differences among the treatments (Figure 2C). The soluble solids content in NB+GJ fruits increased by 12.0% in autumn and 9.8% in spring compared to the GJ group, although these differences were not statistically significant, and no significant variation was observed between the NB+GJ and CK groups (Figure 2D). Both the NB+GJ and GJ groups exceeded the CK group in soluble sugar content, but the spring experimental data did not reach statistical significance (Figure 2E). Notably, fruits from the CK group exhibited higher vitamin C levels compared to the grafted groups (NB+GJ and GJ), particularly in autumn, with increases of 26.2% and 63.3% relative to the NB+GJ and GJ treatments, respectively (Figure 2F).

The synergistic application of NBmelon-1 inoculation and grafting significantly enhances melon yield, average fruit weight, and fruit thickness while maintaining consistent fruit morphology. This combined treatment also increased the soluble solids and soluble sugar content; however, there was no significant difference in vitamin C levels.

### 3.3. Effects of Different Treatments on Rhizosphere Microbial Diversity

**Figure 3:**
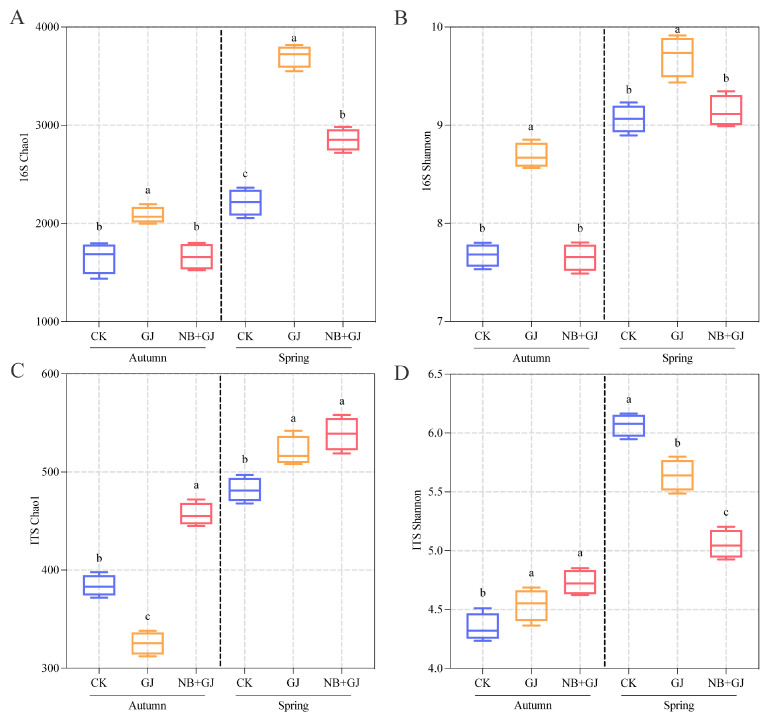
Treatment effects on rhizosphere microbial alpha diversity. (**A**) Bacterial Chao1 index. (**B**) Bacterial Shannon index. (**C**) Fungal Chao1 index. (**D**): Fungal Shannon index. Different lowercase letters denote significant differences (*p* < 0.05).

Rhizosphere soil samples from all treatment groups were sequenced to investigate the treatment-specific impacts on microbial diversity. Bacterial sequencing yielded 13,537 amplicon sequence variants (ASVs) spanning 40 phyla, 105 classes, 227 orders, 316 families, and 434 genera. Alpha diversity analysis revealed that the GJ groups exhibited higher bacterial Chao1 and Shannon indices than the NB+GJ and CK groups across seasons (*p* > 0.05), with only the spring NB+GJ group showing a significantly elevated Chao1 compared to CK (Figure 3A,B). Fungal sequencing identified 2473 ASVs across 15 phyla, 40 classes, 94 orders, 199 families, and 378 genera. In autumn, the NB+GJ group increased fungal Chao1 by 40.4% and 15.9% compared to the GJ and CK groups, respectively, while both grafted groups exhibited higher Shannon indices than CK. In spring, the NB+GJ and GJ groups enhanced fungal Chao1 by 10.6% and 7.5%, respectively, but had lower Shannon indices than CK (Figure 3C,D).

Our results revealed that grafting had a more significant impact on bacterial community structure, while the combined application of NBmelon-1 inoculation and grafting exhibited more pronounced regulatory effects on fungal consortia. Seasonal analysis indicated stronger season-dependent dynamics in microbial abundance and richness.

**Figure 4:**
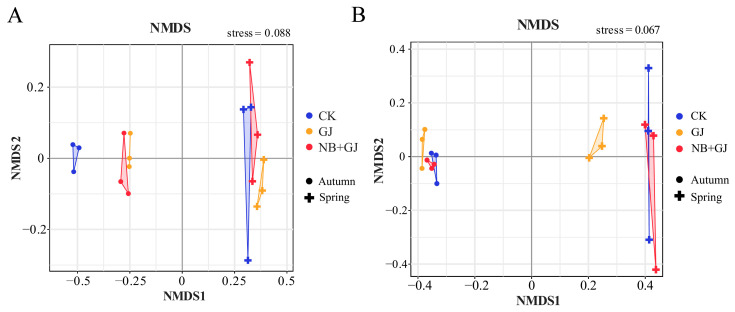
NMDS ordination of melon rhizosphere microbiomes under treatments. (**A**) Bacterial communities. (**B**) Fungal communities. NMDS stress values < 0.1 confirm configuration reliability.

Non-metric multidimensional scaling (NMDS) based on Bray–Curtis distance (stress < 0.1: bacteria = 0.088; fungi = 0.067) demonstrated a significant treatment-driven separation of microbial communities (Figure 4). Distinct separation and compositional differences were observed in the rhizosphere soil microbial communities across seasons, indicating that seasonal variation influences community assembly.

### 3.4. Effects of Different Treatments on Rhizosphere Microbial Community Composition

**Figure 5:**
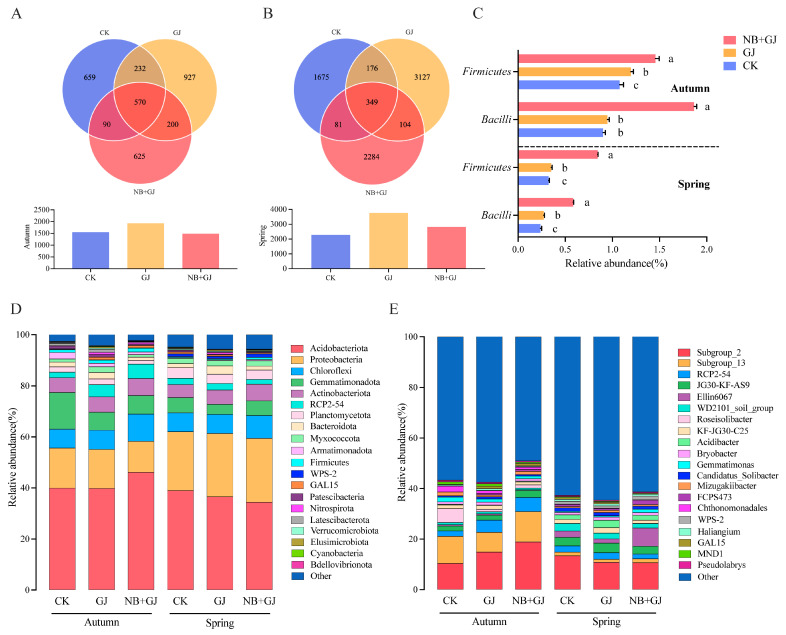
Treatment effects on rhizosphere bacterial community structure. (**A**) Venn diagram of shared bacterial ASVs among treatments in autumn. (**B**) Venn diagram of shared bacterial ASVs among treatments in spring. (**C**) Relative abundance differences of Firmicutes and Bacilli across treatments. Different lowercase letters indicate significant differences (*p* < 0.05). (**D**) Phylum-level composition of bacterial communities under treatments. (**E**) Genus-level composition of bacterial communities under treatments.

Venn diagram analysis revealed significant seasonal divergence in the bacterial communities of the melon rhizosphere (Figure 5). The autumn core bacterial ASVs (*n* = 570) accounted for 29.5% to 38.4% of the total ASVs across the groups, with the NB+GJ, GJ, and CK groups containing 625, 927, and 659 unique ASVs, respectively (Figure 5A). In contrast, the spring core ASVs decreased to 349, representing 9.3% to 15.3% of the total, while the unique ASVs in the NB+GJ, GJ, and CK groups expanded to 2284, 3127, and 1675, respectively (Figure 5B). Comparative analysis demonstrated a 63.3% reduction in unique bacterial ASVs in spring compared to autumn.

Distinct treatment-induced variations in the rhizosphere bacterial communities of melon were observed at both the phylum and genus levels. At the phylum level, the dominant taxa (relative abundance > 5%) included Acidobacteriota (34.35–47.13%), Proteobacteria (12.13–25.06%), Chloroflexi (6.88–10.69%), and Actinobacteriota (5.39–7.79%) (Figure 5D). The relative abundance of Actinobacteriota increased in autumn by 0.90% and 0.20% in the NB+GJ and GJ groups, respectively, with more substantial enhancements of 1.36% and 0.43% observed during spring. Gemmatimonadota exhibited seasonal declines, with reductions of 7.18% (NB+GJ) and 6.74% (GJ) in autumn and 0.16% (NB+GJ) and 1.91% (GJ) in spring compared to the CK group. Notably, NB+GJ treatment significantly enriched both Firmicutes and its class Bacilli across seasons. While the abundance of *Bacilli* in the autumn GJ group showed no significant difference compared to CK, all other treatments demonstrated significant increases in both Firmicutes and *Bacilli* (Figure 5C).

Dominant bacterial genera with a relative abundance greater than 1% included *Subgroup_2* (10.40–18.85%), *Subgroup_13* (6.88–10.69%), RCP2-54 (1.88–5.60%), and JG30-KF-AS9 (1.94–3.77%) (Figure 5E). The grafted groups exhibited contrasting seasonal trends in the abundance of *Subgroup_2*: in autumn, the NB+GJ and GJ groups increased by 8.45% and 4.47%, respectively, compared to the control group (CK), while in spring, these groups decreased by 2.75% (NB+GJ) and 2.61% (GJ). *Subgroup_13* displayed treatment-specific seasonal variations, with NB+GJ showing increases of 1.33% in autumn and 0.09% in spring relative to CK, whereas GJ experienced declines of 2.87% in autumn and 0.24% in spring. The relative abundance of *Subgroup_2* exhibited contrasting seasonal trends in the grafted treatments. In autumn, the NB+GJ and GJ groups showed increases of 8.45% and 4.47% compared to CK, respectively, while in spring, these groups displayed decreases of 2.75% and 2.61% relative to CK. Similarly, *Subgroup_13* demonstrated season-dependent variations: NB+GJ increased by 1.33% and 0.09% in autumn and spring, respectively, compared to CK, while GJ decreased by 2.87% and 0.24% across the seasons. Distinct dominant genera were identified between the seasons. *Roseisolibacter* was predominant in autumn, representing 1.27%, 1.03%, and 5.48% of the microbial communities in the NB+GJ, GJ, and CK groups, respectively. However, its abundance declined to less than 0.06% in all groups during spring. In contrast, Ellin6067 was predominant in spring, accounting for 3.10%, 1.72%, and 2.51% in the NB+GJ, GJ, and CK groups, respectively, while its abundance was less than 0.5% in autumn.

**Figure 6:**
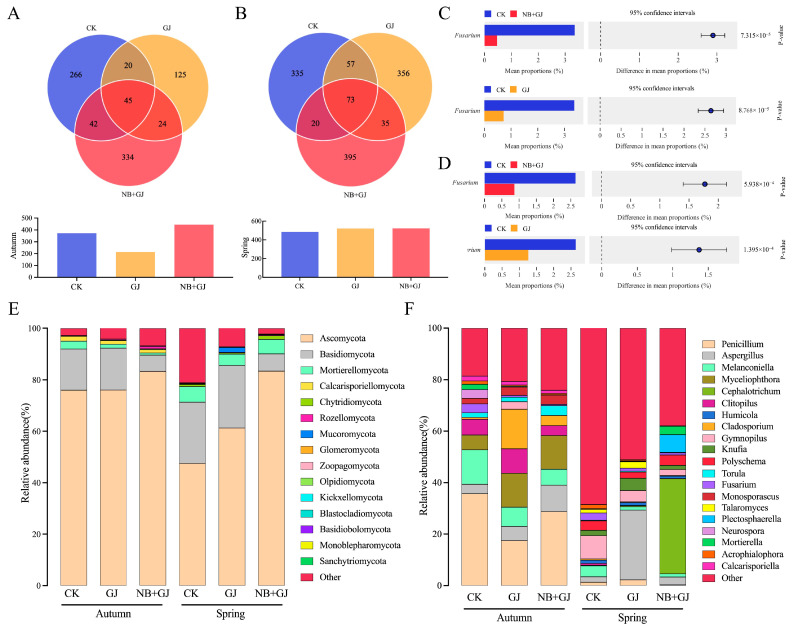
Treatment effects on rhizosphere fungal community structure. (**A**) Venn diagram of shared fungal ASVs among treatments in autumn. (**B**) Venn diagram of shared fungal ASVs among treatments in spring. (**C**) Differential enrichment of Fusarium relative abundance between CK vs. GJ and CK vs. NB+GJ in autumn. (**D**) Differential enrichment of Fusarium relative abundance between CK vs. GJ and CK vs. NB+GJ in spring; (**E**) Phylum-level composition of fungal communities under treatments. (**F**) Genus-level composition of fungal communities under treatments.

Venn diagram analysis revealed significant seasonal dynamics in the fungal communities of the melon rhizosphere (Figure 6). The autumn core fungal amplicon sequence variants (ASVs) (*n* = 45) accounted for 10.1% to 21.0% of the total ASVs, with the NB+GJ, GJ, and CK groups containing 334, 125, and 266 unique ASVs, respectively (Figure 6A). In spring, the number of core ASVs increased by 62.2% (*n* = 73, representing 14.0% to 15.1% of the total), accompanied by a significant expansion of unique ASVs across the groups (NB+GJ: 395, GJ: 356, CK: 335) (Figure 6B).

Significant treatment-driven variations in the fungal communities of the melon rhizosphere were observed at both the phylum and genus levels. The dominant fungal phyla, defined as those with a relative abundance greater than 5%, included Ascomycota (47.51–83.39%), Basidiomycota (6.34–24.33%), and Mortierellomycota (0.82–6.02%) (Figure 6E). Ascomycota consistently maintained its dominance across seasons, with the autumn NB+GJ and GJ groups increasing by 7.30% and 0.10%, respectively, compared to the control group (CK). In spring, enhancements reached 35.89% for NB+GJ and 13.80% for GJ. Basidiomycota exhibited treatment-specific dynamics: the NB+GJ treatment reduced its abundance by 9.58% in autumn and 17.12% in spring compared to CK, while GJ showed no significant seasonal variation. Notably, Calcarisporiellomycota emerged as an autumn-specific phylum, with a relative abundance of 1.20–1.87%, but became negligible (<0.01%) in spring.

Dominant fungal genera included *Penicillium* (0.25–35.71%), *Aspergillus* (1.06–10.27%), and *Melanconiella* (1.20–23.42%) (Figure 6F). *Penicillium* was the predominant genus in the autumn experiment, with a relative abundance ranging from 17.47% to 35.71%. In comparison to the control group (CK), the NB+GJ and GJ groups exhibited an increase in *Aspergillus* abundance, while *Melanconiella* abundance decreased. Both the NB+GJ and GJ treatments significantly reduced the relative abundance of the phytopathogen *Fusarium* compared to CK in both autumn and spring experiments (*p* < 0.05). This indicates that grafting or combined treatment with NBmelon-1 inoculation significantly suppressed the pathogen *Fusarium*, with the NB+GJ intervention demonstrating particularly pronounced effects on the restructuring of the rhizosphere fungal community.

### 3.5. Prediction of Rhizosphere Microbial Function of Melon Under Different Treatments

**Figure 7:**
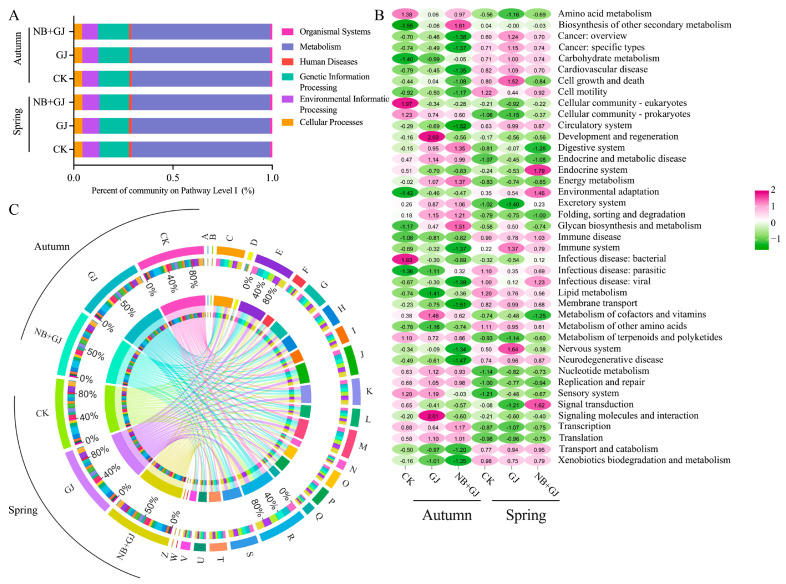
Functional prediction of bacterial communities in melon rhizosphere soil under different treatments. (**A**) KEGG (Level I) functional analysis of rhizosphere bacteria. (**B**) KEGG (Level II) functional analysis of rhizosphere bacteria. (**C**) COG functional analysis of rhizosphere bacteria.

KEGG database annotation revealed a predominance of metabolic functions (69.6–69.9%) in the melon rhizosphere microbiome, followed by Genetic Information Processing (14.8–15.6%), Environmental Information Processing (8.0–8.5%), Cellular Processes (4.1–4.4%), Human Diseases (1.1–1.4%), and Organismal Systems (1.0–1.1%) (Figure 7A). Secondary functional classification identified 41 metabolic pathways (Figure 7B), with Carbohydrate Metabolism, Amino Acid Metabolism, Membrane Transport, and Signal Transduction each constituting over 5% of the relative abundance. Grafting treatments (NB+GJ, GJ) activated carbohydrate metabolism pathways, resulting in abundance increases of 1.2–3.3% compared to the control group (CK) across seasons. Seasonal analysis demonstrated upregulation of amino acid metabolism and membrane transport functions in autumn grafts compared to CK, in contrast to suppression observed in spring. COG functional annotation revealed an enrichment of rhizosphere bacterial genes across 24 functional families, with General Function Prediction (R) exhibiting the highest abundance (Figure 7C). Secondary dominant categories included Amino Acid Transport and Metabolism (E), Translation, Ribosomal Structure, and Biogenesis (J), Cell Wall/Membrane/Envelope Biogenesis (M), and Energy Production and Conversion (C). Grafting treatments (NB+GJ, GJ) slightly downregulated the abundance of categories R, E, J, and M compared to CK, while upregulating Energy Production and Conversion (C).

## 4. Discussion

Continuous cropping presents significant challenges to sustainable melon production, stemming from synergistic interactions between biotic and abiotic factors. These include soil physicochemical degradation, the accumulation of autotoxic compounds, dysbiosis of the rhizospheric microbial community, and the proliferation of soil-borne pathogens and pests [22]. Current mitigation strategies involve optimized cultivation practices, rootstock grafting, soil sterilization, and the application of microbial inoculants, with rootstock grafting recognized as one of the most straightforward and effective interventions given that soil-borne pathogens predominantly invade through root systems [23]. This study employed melon rootstocks to improve graft compatibility and fruit quality, despite their suboptimal root development. To address this limitation, we pioneered the synergistic application of the growth-promoting bacterium *P*. *polymyxa* NBmelon-1 in conjunction with grafting techniques. However, the agronomic efficacy of this integrated approach within melon cultivation systems remains uncharacterized. While previous studies have demonstrated the individual benefits of grafting or microbial inoculation on rhizosphere microbiota, the potential synergistic effects of pre-grafting rhizobacterial inoculation require systematic investigation. This study examined the impacts of combined NBmelon-1 inoculation and grafting on melon development and the structure of the rhizosphere microbial community through an integrated assessment of plant growth, fruit quality metrics, rhizosphere microbial community structure, and functional gene profiles.

*Paenibacillus polymyxa* induces systemic resistance and enhances the stress defense capabilities of plants while promoting root development, ultimately improving overall plant performance [24]. Melon self-rootstock is often selected for grafting due to its genetic compatibility with scions and minimal impact on fruit quality. However, its application is constrained by suboptimal root system development and compromised stress tolerance. Inoculation with *P*. *polymyxa* NBmelon-1 effectively improved root architecture and enhanced overall plant growth performance. This study demonstrated a significant improvement in the morphology of melon self-rootstock through NBmelon-1 inoculation, with notable increases in stem diameter, height, and root length compared to non-inoculated controls. These findings indicate a substantial alleviation of root system vigor deficiencies in melon self-rootstock grafting systems as a result of NBmelon-1 inoculation. Grafting promotes plant growth and yield enhancement by regulating endogenous hormone metabolism and accumulating growth-related metabolites, thereby improving root nutrient absorption efficiency, although fruit quality parameters become dependent on the rootstock [25]. Our findings demonstrate that the combined treatment of NBmelon-1 inoculation and grafting significantly increased fruit yield, average weight, and fruit thickness compared to controls, while maintaining consistent fruit shape. This synergistic approach also elevated the content of soluble solids and soluble sugars; however, there was no significant difference in vitamin C content.

Rhizosphere microorganisms act as the “First Line of Defense” against pathogen invasion in plants, with diverse microbial communities promoting plant growth through both direct and indirect mechanisms [26]. Soil-borne diseases refer to pathogen-driven outbreaks triggered when continuous monoculture of the same crop species accelerates soil pathogen accumulation beyond a critical threshold [27]. Therefore, maintaining microbial diversity emerges as a key strategy for suppressing soil-borne diseases. However, the diversity of rhizosphere microorganisms is influenced by multiple factors, primarily biological determinants (such as plant genotype and developmental stage) and abiotic factors (including seasonal variations, climatic conditions, and agricultural practices) [28]. For instance, grafting enhances rhizosphere microbial diversity, with increased niche overlap among microbial communities competitively excluding pathogens by occupying shared ecological niches [14]. This study revealed that various treatments enhanced the richness and diversity of rhizosphere bacterial communities. In contrast, fungal communities exhibited more complex dynamic patterns. During spring, both treatment groups showed increased fungal richness but reduced diversity in the rhizosphere. Conversely, in autumn, the NB+GJ treatment group exhibited significantly increased rhizosphere fungal richness and diversity indices compared to the control group (CK), while the GJ treatment group demonstrated reduced fungal richness but enhanced diversity. Seasonally driven rhizosphere microbial diversity with elevated autumn temperatures and increased precipitation-enhancing microbial metabolic activity was shown to promote pathogen proliferation, potentially explaining the intricate restructuring of fungal communities. Temporal analysis revealed greater microbial richness and diversity in spring compared to autumn, indicating significant seasonal modulation of rhizosphere microbial assemblages.

Distinct structural profiles emerged among treatment groups in rhizosphere microbial communities under non-sterile conditions. For example, 16S rRNA sequencing revealed significant increases in the relative abundance of Acidobacteriota and Firmicutes within the grafted groups (NB+GJ, GJ), with a more pronounced enhancement observed in the NB+GJ treatment. Acidobacteriota, recognized as archetypal antagonistic taxa, suppress phytopathogens through the secretion of antimicrobial metabolites and nutrient competition [29,30,31]. Firmicutes contribute to host defense by stimulating plant systemic resistance and maintaining physiological homeostasis [32]. Seasonal variations similarly drive structural shifts in rhizosphere microbial communities, with distinct microbial taxa potentially attaining dominance during specific seasons [33]. Acidobacteriota, a beneficial bacterial phylum, exhibited a marginal increase in the autumn-grafted groups (NB+GJ, GJ) but declined in the spring-grafted counterparts, demonstrating seasonal modulation of rhizobacterial community architecture. *Paenibacillus polymyxa* belongs to the Firmicutes phylum, specifically within the Bacilli, Bacillales, Paenibacillaceae, and *Paenibacillus* genera. The combined treatment of NBmelon-1 inoculation and grafting significantly elevated the relative abundance of *Bacilli*, confirming the sustained high colonization activity of this strain in rootstock-grafting composite systems. Ascomycota, a predominant fungal phylum, plays a pivotal role in nutrient cycling by secreting diverse enzymes capable of decomposing recalcitrant organic substrates such as lignin [34]. Its relative abundance showed consistent enhancement across grafted groups, facilitating nutrient uptake by plants. Notably, the combination of NBmelon-1 inoculation and grafting treatments significantly reduced the relative abundance of phytopathogens, including *Melanconiella* and *Fusarium*. Previous studies have identified *Melanconiella* as a critical plant pathogen, while *Fusarium* is the primary causal agent of melon *Fusarium* wilt, one of the most susceptible and lethal soil-borne phytopathogens [35,36]. This study demonstrates that the synergistic application of NBmelon-1 inoculation and grafting enhances the enrichment of antagonistic bacterial communities, suppresses pathogenic fungal proliferation, and drives a structural transition in rhizosphere microbiota from fungal-dominated to bacterial-dominated profiles, thereby establishing optimized environmental conditions for host plant growth.

Structural shifts in rhizosphere microbial communities may influence their functional composition. Consequently, functional gene profiling of rhizosphere microbiota enables predictive assessments of their metabolic dynamics, providing critical insights into plant-microbe–soil interactions [37,38]. KEGG annotation identified metabolic pathways as the functional cornerstone of rhizosphere microbiota, which play a crucial role in governing plant developmental processes. Secondary functional hierarchies were primarily characterized by carbohydrate metabolism, amino acid metabolism, membrane transport, and signal transduction. Notably, the combined inoculation of NBmelon-1 with grafting (NB+GJ) significantly increased the abundance of carbohydrate metabolism pathways, suggesting that the synergistic application of plant growth-promoting rhizobacteria (PGPR) inoculation and grafting enhances microbial efficiency in carbohydrate decomposition, transformation, and subsequent nutrient provisioning [39]. Functional annotation using the COG database classified melon rhizosphere microbiomes into 24 functional categories, with general functional prediction and amino acid transport/metabolism being critical for melon morphogenesis. Comparative analysis revealed significant seasonal fluctuations in rhizosphere functional abundance, demonstrating the microbial community’s responsiveness to environmental changes.

## 5. Conclusions

Field trials demonstrated that inoculation with *P*. *polymyxa* NBmelon-1 significantly enhanced stem diameter and root growth in melon rootstock. This treatment simultaneously increased the yield of grafted scions, individual fruit weight, and pulp thickness without altering fruit morphology. Compared to non-grafted controls (CK), the synergistic inoculation-grafting treatment elevated soluble solids and sugar content in the fruits, although it reduced vitamin C levels. High-throughput sequencing revealed that this synergistic application enriched the rhizosphere microbial diversity, selectively amplified beneficial microbiota, and strengthened systemic pathogen resistance. Seasonal variations profoundly influenced both the diversity and taxonomic composition of rhizosphere communities. Collectively, NBmelon-1 inoculation mitigates root system limitations in melon rootstock grafting, improves fruit yield and quality metrics, and enhances disease resistance through targeted microbial community restructuring. This study provides novel insights into optimizing melon self-rootstock grafting and alleviating the challenges of continuous cropping. Future research should elucidate the mechanisms by which beneficial microbiota, enriched in melon-grafted seedlings under the synergistic effects of NBmelon-1 inoculation and grafting, suppress soil-borne pathogens.

## Data Availability

The original contributions presented in this study are included in the article. Further inquiries can be directed to the corresponding author.

## Appendix A

**Table A1:** Graft survival rate of different treatments.

Treatment	Autumn (%)	Spring (%)
NB+GJ	96.0	98.5
GJ	85.0	82.5

**Table A2:** Total yield of melon under different treatments.

Treatment	Autumn (kg)	Spring (kg)
NB+GJ	65.83	67.02
GJ	54.04	61.92
CK	50.71	49.08
